# Determination of Epiphyseal Union Age in the Knee and Hand Joints Bones among the Saudi Population in Taif City

**DOI:** 10.1155/2018/7854287

**Published:** 2018-05-20

**Authors:** Majed O. Aljuaid, Osama R. El-Ghamry

**Affiliations:** Medical College, Taif University, Taif 21974, Saudi Arabia

## Abstract

**Introduction:**

The use of radiographic data for determination of age, according to the epiphyseal union stage, is a widely accepted method and considered scientifically approved. The aim of the present work is to estimate the age of epiphyseal union of hand and knee joints bones among Saudi population in Taif City.

**Subjects and Method:**

A retrospective cross-sectional study was conducted in the Armed Forces Hospitals (4 hospitals) in Taif City. The five-stage method was used for the union assessment.

**Results:**

A total of 473 patients' X-ray images were involved. Approximately three-quarters of the knee and hand images were males' images (77.25% and 75.41%, resp.). The means of age of stage 3 (age of recent union) in the knee joint were 23.63 ± 3.12 and 21.19 ± 3.41 in males and females, respectively, and 19.84 ± 3.47 and 17.19 ± 1.61 in hand joints for males and females, respectively. There were significant differences between males and females in the means of age for stages 1, 3, and 4 at the knee joint plates and for stages 0, 2, and 3 in the hand joint plates (*P* values were <0.05). However, by comparing the mean of age for each stage with the previous stage mean in males and females, there was a significant difference between stages 0–4 (*P* values ≤ 0.0001, ≤0.001, <0.0001, and <0.001, resp.) and stages 2 and 3 (*P* value = 0.012) in knee joint images for males and females, respectively. In addition, there were significant differences between stages 2–4 in hand joints for males and females (*P* values ≤ 0.0001, <0.0001, <0.0001, and <0.0001, resp.) and stages 0 and 1 for males only (*P* value = 0.002).

**Conclusion and Recommendation:**

This study suggests that the union of epiphyses of knees and hands in Taif City occurs later than other places. More studies must be done with female samples.

## 1. Introduction

Determination of human chronological age was extensively investigated by biological anthropologists, archeologists, and anatomists from many perspectives, one of which was the epiphyseal union and its relationship to chronological age [1–3]. This approach used the archeological (autopsied) skeletal collection [2, 4, 5], although radiographic data was used instead, as it is easier to obtain a greater number of images in each phase (of early ages) of the study's population; the epiphyseal union stages are confined to this era of human life [1, 6]. The use of radiographic data for determination of age according to epiphyseal union stage is considered to be scientifically approved [7–9]. The study group on forensic age diagnostics recommended a physical examination, X-ray of the left hand, and a dental examination to determine the age of young people (before epiphyseal union) [10, 11].

The age at each stage of an epiphyseal union is a parameter known to be multifactorial [3, 4, 7, 12–17], which will consequently affect the process of the union at the epiphyses. The differences in geographical distribution, socioeconomic status, climate, metabolism, nutrition, and genetics (race) comprise some of these factors. Also, ethnicity is one of the main reasons for this kind of study with a neglected population in this field [3, 4, 7, 12–17]. The application for this project in a clinical situation, a forensic investigation, and medicolegal aspects has a number of dimensions. In the forensic investigation, we are determining chronological age accurately in cases of young, unknown corpses. Clinically, medical consultation in cases of incorrect dating of birth or absent date of birth in young refugees is necessary [6, 8, 18–20]. On the other hand, it also determines chronological age for judicial responsibility of young people who commit a crime, but do not have an identity. Establishment of accurate linkage between biological and chronological age is used in practical situations mentioned above. The epiphyses are the growth plates for the long bones. The aim of this study was to determine the age of the epiphyseal union in the knee and hand joints of the Saudi population with X-ray images, including males and females. Thus, the primary outcome of this study will identify the ages of different epiphyseal union stages in males and females. The secondary outcome will be the difference between male and female samples of different epiphyseal union stages. This study will concentrate on two areas of the body: the knee and hand. Selecting these areas include a high number of images, largely due to trauma, as young people are more prone to engage in dangerous activities. In this study, the revised method for assessment of the union of the epiphysis was used [1, 17]. This method was first outlined by McKern et al. in 1957, who divided the epiphyseal union into five stages: nonunion, beginning of union, active union, recent union, and complete union [1, 17]. McKern et al. worked on osseous specimens of the knee joint, first applied via knee radiographic image assessment by O'Connor and colleagues [1]. This method was also applied on hand joint radiographic image assessments by Bhise et al. [17].

## 2. Methods

The current study is retrospective and cross-sectional, including all patients who have X-ray images over the last 6 years (between the end of July 2010 and July 2016) in the Armed Forces Hospitals (4 hospitals) in Taif City; this fulfilled inclusion criteria and was kept separate from exclusion criteria. Inclusion criteria included images of Saudi patients, between 4 and 30 for females and 6 and 32 for males, who lived in Taif since birth; they must also verify their date of birth and have X-rays with available reports and radiographic images at the Armed Forces Hospitals. Exclusion criteria were fracture of the epiphysis, congenital anomaly of the bones, and/or bone disease. Data was collected from patients' electronic health records onto an Excel sheet with a hospital computer, thereby maintaining any nominative information: patients were identified by a serial study code and initials. These were linked to patients' names and medical record numbers (MRNs) on a separate identification log sheet, kept in a locked location. After verification, data were transferred to the statistical database with SPSS (Statistical Package for the Social Sciences) program v. 20. The X-ray reports for these patients were examined to separate them from exclusion criteria. Patient files were examined in both the presence of exclusion criteria and in the presence of a defective report, or else the case was excluded. Those patients were statistically investigated. The IRB (Institutional Review Board) for both hospitals and the University Ethical Committees approved the waiver of the informed consent, as this was a retrospective study.

### 2.1. The Method of Assessment of the Epiphyseal Union

In the assessment of radiographic images of the knee and hand joints, the revised method for epiphyseal union assessment was used [1, 17]. The bones of the knee joint that had been included were the lower part of the femur and the upper part of the tibia and fibula. The bones of the hand joints that had been included were the metacarpals and phalanges without the distal ulna, radius, or carpals. If there were different stages in the same radiographic image, we considered the last one. This approach was used to obtain the age of union for the joint bones as a whole without segregation.

**Figure 1:**
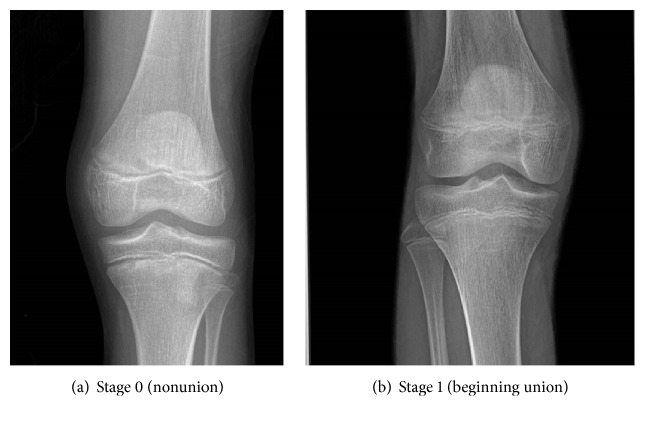
Radiographic images of knee joint showing stage 0 labeled as (a) and stage 1 labeled as (b).

**Figure 2:**
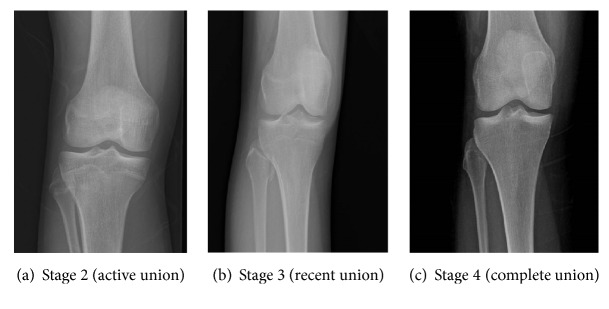
Radiographic images of knee joint showing stage 2 labeled as (a) and stage 3 labeled as (b) and stage 4 labeled as (c).

**Figure 3:**
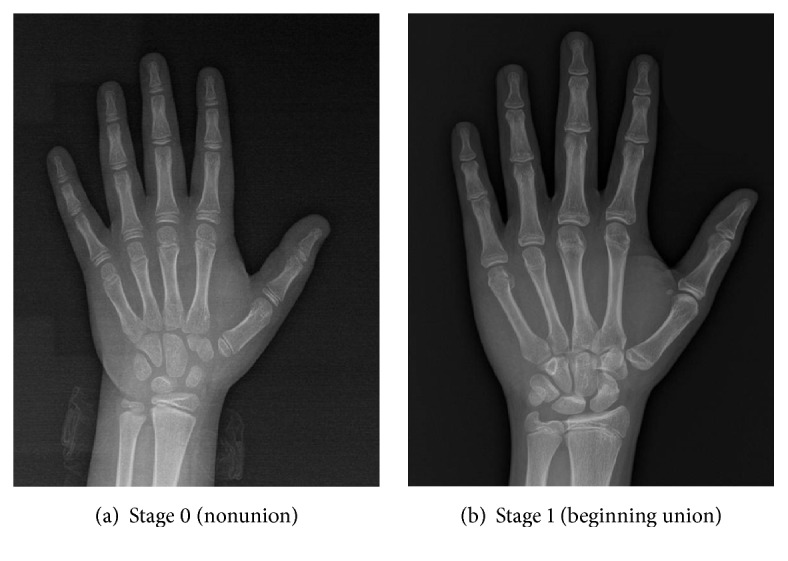
Radiographic images of hand joints showing stage 0 labeled as (a) and stage 1 labeled as (b).

**Figure 4:**
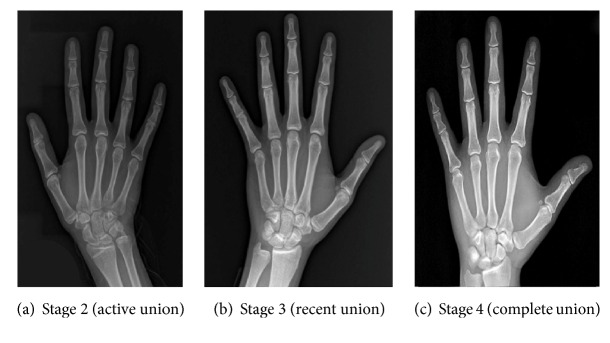
Radiographic images of hand joints showing stage 2 labeled as (a) and stage 3 labeled as (b) and stage 4 labeled as (c).

In stage 0, a radiolucent area was observed between the epiphysis and the diaphysis (Figure 1). In stage 1, there is a continuous radiolucent gap between the epiphysis and diaphysis, which is separable at the extremes (Figure 1). In stage 2, a radio-opaque thick line was observed between the epiphysis and diaphysis (Figure 2). In stage 3, a continuous radiopaque thin line was observed, called the union scar (Figure 2). In stage 4, the union scar disappeared and both the epiphysis and diaphysis appeared fused as one bone (Figure 2). Similarly, stages 0–4 on hand plates were assessed in the same manner (Figures 3 and 4). Moreover, X-rays showed more advanced results in the active union stage compared to the macroscopic results; this showed less advanced results in the complete union stage [2].

After deleting the patient data on the images, hand and the knee joints were assessed. Training for this method was done by our mentor (Professor Osama Elghamri).

### 2.2. Statistical Analysis

All continuous variables were expressed as mean ± SD. Categorical variables were compared with the *X*^2^ test and *t*-test for comparing means of continuous variables. A *P* value must be less than 0.05 to be considered significant, except for the correlation coefficient (spearman's rho) and linear regression in which the *P* value must be less than 0.01 to be considered significant. Linear regression was used to detect the best fitting line between the stages of epiphyseal union and the exact age of cases. This method will analyze the predictability of the relationship between the two variables (age and stage). Also, linear regression was used to calculate the regression equation to predict the age of a case with the stage number of involved cases.

## 3. Results

**Figure 5:**
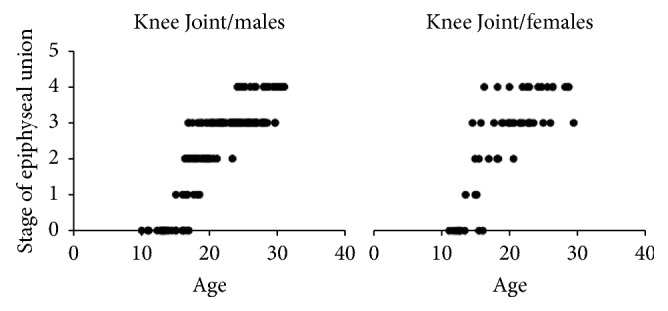
The graphical presentation of our sample range of age in the different epiphyseal union stages in knee joint.

**Figure 6:**
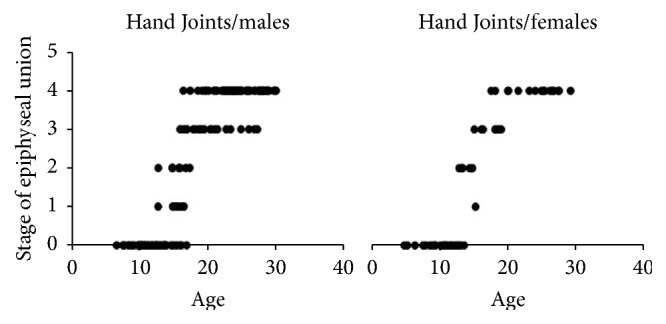
The graphical presentation of our sample range of age in the different epiphyseal union stages in hand joints.

**Figure 7:**
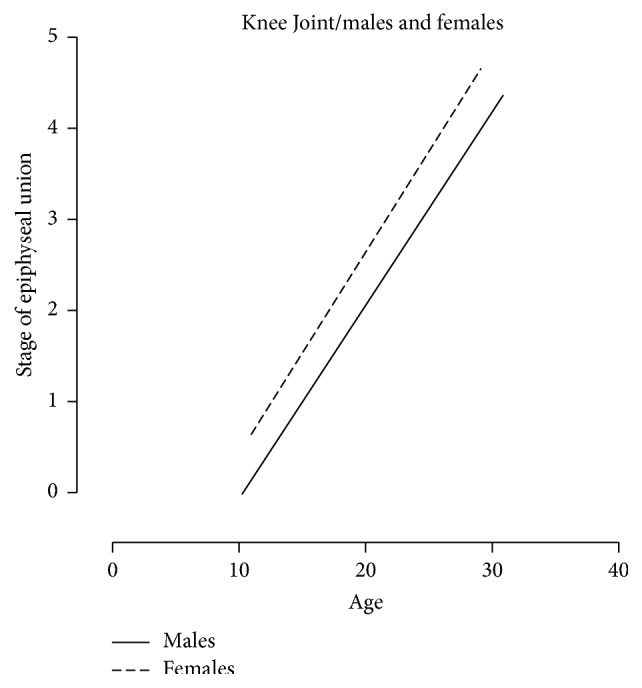
The graphical presentation of correlation best fit line between the age and stages of epiphyseal union in knee joint.

**Figure 8:**
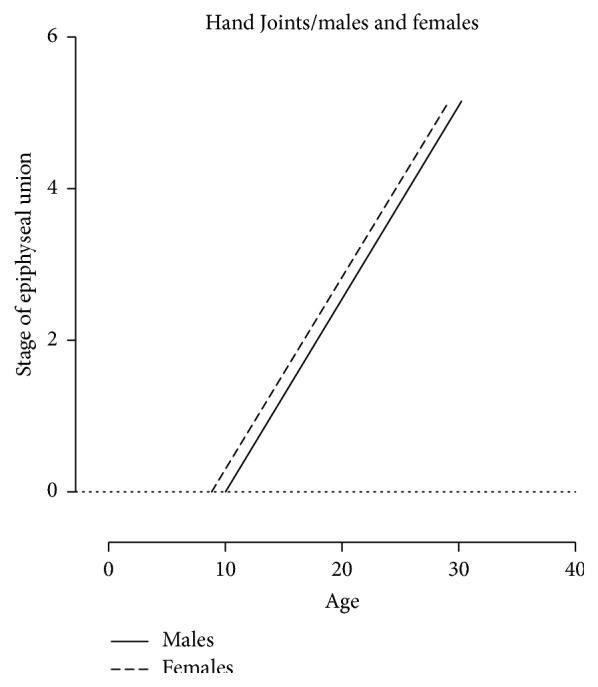
The graphical presentation of correlation best fit line between the age and stages of epiphyseal union in hand joints.

A total of 473 patients' X-ray images (*N* = 473) was involved in this retrospective cross-sectional study. Approximately half of this sample involved knee joint images (*n*1 = 233) while the other half was hand images (*n*2 = 240). Approximately three-quarters of the knee images and hand images were male images (77.25% and 75.41%, resp.). The number of the knee and hand images for each age stage interval of the epiphyseal union in each gender is shown in Tables 1 and 2. Patients' ages ranged from 4 to 32 years, with a different means of age in each stage, as shown in Table 3. The epiphyseal union in the knee joint was first recorded (stage 1: beginning of union) at age 13 in the female group and at age 15 in the male group (Figure 5). However, the means of the age in stage 1 were 16.77 ± 1.24 and 14.51 ± 0.87 in males and females, respectively. On the other hand, the hand joints had started the epiphyseal union at age 12 in the male group. Nevertheless, at age 12 in the female group, there was an active union case, indicating that the beginning of the union of hand joints in the female group was younger than the male group (Figure 6). Recent completion of epiphyseal union (stage 3) at the knee joint was noted at age 14 in the female group and at age 16 in the male group (Figure 5). United epiphyses of the hand joints were observed at the age of 16 years in the male group and 15 years in the female group (Figure 6). Stage 3 means of age in the knee joint included 23.63 ± 3.12 and 21.19 ± 3.41 in males and females, respectively; in hand joints, it was 19.84 ± 3.47 and 17.19 ± 1.61 in males and females, respectively. Correlation coefficients (spearman's rho) were 0.851 and 0.887 for knee and hand joints, respectively, in males (*P* value ≤ 0.0001) and 0.752 and 0.901 in females (*P* value ≤ 0.0001), and the best fit line for the correlation between age and the epiphyseal union of knee and hand joints is depicted in Figures 7 and 8, respectively. There were significant differences between males and females in the means of age for stages 1, 3, and 4 at knee joint plates and for stages 0, 2, and 3 at hand joint plates (*P* values shown in Table 3). However, by comparing the means of age for each stage with the previous stage means in males and females, there was a significant difference between stages 0 and 4 (*P* values ≤ 0.0001, 0.001, <0.0001, and <0.0001, resp.) in a male group of knee joint images. Significant differences between the means of age stages 2 and 3 in the female group of knee joint images were observed (*P* value = 0.012). In addition, there were significant differences between stages 2 and 4 in hand joints for males and females (*P* values ≤ 0.0001, <0.0001, <0.000, and <0.0001, resp.). Similarly, male group images of the hand joints showed significant differences between stages 0 and 1 (*P* value = 0.002). Otherwise, there was no significant difference found by comparing means of age of each stage with the previous one. A regression formula was calculated, as shown in Table 4.

## 4. Discussion

In the literature, there were many studies conducted on different parts of the body using various methods and modalities. Also, there were a few studies that used the radiological approach compared to that used for bone remains [1, 3, 6–20]. These studies were done on different populations in race, ethnicity, or residency, which had an influential but comparative effect on the epiphyseal union and on the results [8, 12]. The variation in the ages of epiphyseal union stages between different ethnic groups was the main purpose for previous studies, which ended up demonstrating that there is indeed variation between different ethnic groups [3, 4, 7, 12–17]. The socioeconomic status was one of the factors that influenced the epiphyseal union process [21]. The same author suggested that previous factors do not actually affect this process [21].

However, by reviewing previous study results regarding age of union, we found a wide range [14–23] for knee joint plate unions [11–18] and for hand joint plate unions [1, 9, 15–18, 20–23]. Some previous studies are summarized in Table 5. This variation mandates a wider range of ages in the samples selection process. We will conduct a 7-year margin for the lowest and oldest ages to accurately determine the initiation of union at the completion of the recent union stage. During analysis of results, we extended the latter margin by 2 years (with a year by year approach) to determine the end of stage 3. As a result of this change, the range of sample ages was 4–32 years. This range was used according to the available data in different groups and different areas.

Determination of the union age for each epiphyseal plate was done in previous studies, revealing that the knee and hand joint plates showed the same union stage [1, 17]. Other studies showed that different plates were in different stages; based on this study, there is variation of union age for different plates [1, 7, 8]. This study dealt with plates in hand and knee joints as a whole, without segregation. The relationships between the chronological age of the patient at the time of radiological examination and the stages in males and females were used to determine the most accurate method of union age. This study showed a stronger correlation in the hand joint bones for males and females, compared to knee joint bones. However, a stronger relationship indicates a more accurate method to determine union age; bilateral asymmetry could not be examined in this study due to scarcity of radiographic images depicting both limbs. The right and left discrepancy in the epiphyseal union was not observed in a previous study using the same staging method. However, this could not be examined in the present study.

There were different methods used in previous studies to determine union age. One of these methods considered the age at which more than 50% of the group was completely fused [24]. However, other methods used 85% and 100% as cut-off points [25, 26]. These studies examined different staging methods from those used in this study. Thus, stages 3 and 4 were considered to be united plates (stage 3 in the old classification). Accordingly, applying any approach of the previously mentioned studies must include both stages as one pool. The first method was used in this study to minimize falsified results that could create unequal distribution of images and absence of them at certain ranges. The ages of union were 24 and 21 for knee joints (males and females, resp.), while hand joints were united at 23 and 21 for males and females, respectively. Our findings fell in the range of the previous studies, using the same staging method [1, 17]. In this study, the nonunion of epiphyses was observed at 17 and 16 in knee joints for males and females, respectively, although it was 17 and 13 in hand joints for males and females. These findings are not surprising, compared to documented cases in previous studies, as they revealed older cases with nonunion as well [1].

Age means of males and females in each stage of knee and hand joint bones were statistically examined and regression formulae were calculated to predict age based on images that could be used in many fields (previously mentioned). The insignificant differences were seen between males and females in stages 0 and 2 in the knee joint and stage 2 and 4 in hand joints. These findings could be justified by the discrepancy between the two groups, regarding sample sizes in those stages in this retrospective study, which is limited to available images. However, the gender difference found in the other stages was expected and it confirmed what was revealed in other studies [1]. The males display delayed epiphyseal union compared to females in knee and hand joints. The means of differences between them were 2.25 and 1.5 in hand and knee joint bones [1]. These findings were linear to what other studies had found: 1.5 in the knee joint in one study and 2 in same joint in another. In addition, the means of age in each stage was significantly different from the means of the previous stages in males and females, except in stages 0 and 1 and stages 1 and 2 in hand and knee joints among females. Also, stages 3 and 4 in the knee joint among females and stages 1 and 2 in the hand joint among males were insignificantly different. These findings are justified by the discrepancy of sample numbers at compared stages (Table 3). The same condition was discussed in a previous study, using the same staging method and reviewing the study's sample numbers at each stage: it was the same statistical issue [1].

In the process of determination of sample age, we faced some difficulties that could be seen from three perspectives. First was the absence in the previous study of the same environments or even nearby areas, such as gulf countries. Second, the presence of extensive data was widely disparate regarding the age of union. Third, previous studies' methods and sample types (osseous versus radiological) were widely varied.

## 5. Conclusion and Recommendations

This study suggests that the union of epiphyses of knee and hand in the Saudi population in Taif City occurs later than in other locations. The application of this project in estimating age could be used by many fields, being the ultimate goal the authors have in our own society. It is expected to come across some difficulties in the first time, which will be resolved with more investigations. Future projects in other cities and environments in the same country are being recommended to find a valid formula. We also suggest a greater number of studies with more female samples.

## Figures and Tables

**Table 1:** Numbers of images at each stage of epiphyseal union of the knee joint bones.

Age interval	*n*	Staging of knee epiphyses (males)					*n*	Staging of knee epiphyses (females)				
		0	1	2	3	4		0	1	2	3	4
4–4.99	-	-	-	-	-	-	-	-	-	-	-	-
5–5.99	-	-	-	-	-	-	-	-	-	-	-	-
6–6.99	-	-	-	-	-	-	-	-	-	-	-	-
7–7.99	-	-	-	-	-	-	-	-	-	-	-	-
8–8.99	-	-	-	-	-	-	-	-	-	-	-	-
9–9.99	-	-	-	-	-	-	-	-	-	-	-	-
10–10.99	2	2	-	-	-	-	-	-	-	-	-	-
11–11.99	1	1	-	-	-	-	2	2	-	-	-	-
12–12.99	7	7	-	-	-	-	4	4	-	-	-	-
13–13.99	8	8	-	-	-	-	2	1	1	-	-	-
14–14.99	4	4	-	-	-	-	3	-	1	1	1	-
15–15.99	3	2	1	-	-	-	4	1	1	1	1	-
16–16.99	12	5	4	2	1	-	3	1	-	1	-	1
17–17.99	11	1	1	7	2	-	1	-	-	-	1	-
18–18.99	12	-	2	5	5	-	5	-	-	2	1	2
19–19.99	11	-	-	9	2	-	4	-	-	-	3	1
20–20.99	14	-	-	3	11	-	4	-	-	1	3	-
21–21.99	15	-	-	1	14	-	4	-	-	-	3	1
22–22.99	4	-	-	-	4	-	4	-	-	-	2	2
23–23.99	10	-	-	1	9	-	2	-	-	-	2	-
24–24.99	13	-	-	-	10	3	3	-	-	-	1	2
25–25.99	12	-	-	-	11	1	2	-	-	-	1	1
26–26.99	14	-	-	-	11	3	2	-	-	-	-	2
27–27.99	6	-	-	-	6	-	-	-	-	-	-	-
28–28.99	13	-	-	-	7	6	3	-	-	-	-	3
29–29.99	4	-	-	-	2	2	1	-	-	-	1	-
30–30.99	3	-	-	-	-	3	-	-	-	-	-	-
31–31.99	1	-	-	-	-	1	-	-	-	-	-	-

Sum	180	30	8	28	95	19	53	9	3	6	20	15

**Table 2:** Numbers of images at each stage of epiphyseal union of the hand joints bones.

Age interval	*n*	Staging of hand epiphyses (males)					*n*	Staging of hand epiphyses (females)				
		0	1	2	3	4		0	1	2	3	4
4–4.99	-	-	-	-	-	-	1	1	-	-	-	-
5–5.99	-	-	-	-	-	-	1	1	-	-	-	-
6–6.99	1	1	-	-	-	-	-	-	-	-	-	-
7–7.99	3	3	-	-	-	-	2	2	-	-	-	-
8–8.99	5	5	-	-	-	-	2	2	-	-	-	-
9–9.99	4	4	-	-	-	-	3	3	-	-	-	-
10–10.99	12	12	-	-	-	-	10	10	-	-	-	-
11–11.99	6	6	-	-	-	-	5	5	-	-	-	-
12–12.99	14	12	1	1	-	-	7	6	-	1	-	-
13–13.99	13	13	-	-	-	-	3	1	-	2	-	-
14–14.99	8	5	1	2	-	-	2	-	-	2	-	-
15–15.99	6	3	2	1	-	-	2	-	1	-	1	-
16–16.99	12	2	2	2	6	-	2	-	-	-	2	-
17–17.99	7	2	-	1	2	2	1	-	-	-	-	1
18–18.99	5	-	-	-	4	1	4	-	-	-	3	1
19–19.99	7	-	-	-	4	3	1	-	-	-	-	1
20–20.99	3	-	-	-	2	1	1	-	-	-	-	1
21–21.99	9	-	-	-	2	7	1	-	-	-	-	1
22–22.99	8	-	-	-	1	7	-	-	-	-	-	-
23–23.99	8	-	-	-	-	8	2	-	-	-	-	2
24–24.99	9	-	-	-	-	9	1	-	-	-	-	1
25–25.99	10	-	-	-	1	9	2	-	-	-	-	2
26–26.99	6	-	-	-	1	5	3	-	-	-	-	3
27–27.99	8	-	-	-	2	6	2	-	-	-	-	2
28–28.99	10	-	-	-	-	10	-	-	-	-	-	-
29–29.99	5	-	-	-	-	5	1	-	-	-	-	1
30–30.99	2	-	-	-	-	2	-	-	-	-	-	-
31–31.99	-	-	-	-	-	-	-	-	-	-	-	-

Sum	181	68	6	7	25	75	59	31	1	5	6	16

**Table 3:** Mean, standard deviation (SD), range of age (years), and significance of difference between males' and females' ages at each stage of epiphyseal union of the knee joint and hand joints bones.

Site	Stage	Males					Females					Sig.
		*n*	Min	Max	Mean	SD	*n*	Min	Max	Mean	SD	
Knee	0	30	10.04	17.01	13.7542	1.72372	9	11.09	16.04	13.0457	1.67232	0.240
	1	8	15.08	18.54	16.7736^*∗*^	1.24244	3	13.51	15.13	14.5105	.87442	0.012^*∗∗*^
	2	28	16.42	23.38	18.8349^*∗*^	1.49106	6	14.87	20.55	17.3662	2.07975	0.062
	3	95	16.89	29.65	23.6318^*∗*^	3.12421	20	14.51	29.38	21.1886^*∗*^	3.40694	0.003^**∗***∗*^
	4	19	24.13	31.02	27.8489^*∗*^	2.26147	15	16.23	28.67	23.4716	3.96549	0.001^*∗∗*^

Hand	0	68	6.65	17.01	12.0113	2.35966	31	4.67	13.50	10.3398	2.09470	<0.001^**∗***∗*^
	1	6	12.78	16.61	15.2434^*∗*^	1.35211	1	-	-	-	-	0.969
	2	7	12.81	17.43	15.6543	1.63450	5	12.77	14.72	13.6455	.83013	0.031^*∗∗*^
	3	25	14.85	27.42	19.8365^*∗*^	3.47392	6	15.01	18.96	17.1913^*∗*^	1.61258	0.038^*∗∗*^
	4	75	16.53	30.18	24.6715^*∗*^	3.22954	16	17.50	29.21	23.9502^*∗*^	3.56247	0.395

*∗* indicates that the mean of the marked stage is significantly (*P* value < 0.05) different from the mean of the previous stage; **∗∗** indicate that the male group mean of the marked stage is significantly (*P* value < 0.05) different from the mean of the female group of the same stage.

**Table 4:** Regression formulae for male and female groups in knee and hand joints.

Epiphyseal union	Regression formula in males	Sig.	Regression formula in females	Sig.
Knee joint	13.203 + 3.463^*∗*^ the least stage	**<0.001** ^*∗*^	12.726 + 2.715^*∗*^ the least stage	**<0.001** ^*∗*^
Hand joints	11.792 + 3.128^*∗*^ the least stage	**<0.001** ^*∗*^	10.042 + 3.202^*∗*^ the least stage	**<0.001** ^*∗*^

**∗** indicates that the *P* value was <0.05.

**Table 5:** Some of the previous studies that were done at the knee and hand regions.

Author	Year	Population	Number of sample	Number of stages	Knee joint	Hand joints
Washburn [22]^*∗*^	1958	US	450	5	Males: 22-23	-* *-* *-* *-* *-* *-* *-* *-* *-* *-
Garn et al. [21]	1961	US	107	3	-* *-* *-* *-* *-* *-* *-* *-* *-* *-	Males: 15.9–16.4 Females: 13.6–14.6
Schaefer and Black [23]^*∗*^	2005	Bosnian	114	5	Males: 17–20	-* *-* *-* *-* *-* *-* *-* *-* *-* *-
O'Connor et al. [1]	2008	Irish	234	5	Males: 17–18.9 Females: 17–17.9	-* *-* *-* *-* *-* *-* *-* *-* *-* *-
Bhise et al. [17]	2011	Indian	299	5	-* *-* *-* *-* *-* *-* *-* *-* *-* *-	Males: 16–18 Females: 15–17

*∗* indicates the use of bone remains instead of imaging studies; the studies were chronologically ordered.
